# Phase Angle Shows a Negative Correlation With Serum GDF‐15 Concentrations in Hospitalized Patients With Cardiovascular Disease

**DOI:** 10.1155/jnme/3379141

**Published:** 2026-07-08

**Authors:** Taira Fukuda, Kan Kondo, Riichi Nishikawa, Rina Hirai, Hayato Ishizaka, Hideaki Tan, Naohiro Nozawa, Seiko Tokoi, Suguru Hirose, Suomi Yamaguchi, Hiroshi Yagi, Takuo Arikawa, Hirohisa Amano, Masashi Sakuma, Ikuko Shibasaki, Hirotsugu Fukuda, Shigeru Toyoda, Toshiaki Nakajima

**Affiliations:** ^1^ Department of Health and Nutrition, University of Human Arts and Sciences, Saitama, Japan, human.ac.jp; ^2^ Department of Cardiovascular Medicine, Dokkyo Medical University School of Medicine, Tochigi, Japan, dokkyomed.ac.jp; ^3^ Department of Rehabilitation, Dokkyo Medical University Hospital, Tochigi, Japan, dokkyomed.ac.jp; ^4^ Department of Cardiovascular Surgery, Dokkyo Medical University School of Medicine, Tochigi, Japan, dokkyomed.ac.jp; ^5^ Department of Medical KAATSU Training, Dokkyo Medical University, Tochigi, Japan, dokkyomed.ac.jp

## Abstract

**Background:**

Growth differentiation factor (GDF)–15 is associated with various conditions such as cardiovascular disease, inflammation, and chronic kidney disease. Phase angle (PhA) reflects cellular health and nutritional status. However, the relationship between serum GDF‐15 concentrations and PhA is unclear.

**Methods:**

Serum GDF‐15 concentrations in patients with heart failure (*n* = 91), aortic stenosis patients undergoing aortic valve replacement (AVR) or transcatheter aortic valve implantation (TAVI) (*n* = 48), and healthy older individuals (*n* = 73) were measured via enzyme‐linked immunosorbent assay. PhA was measured with an impedance‐based body composition analyzer.

**Results:**

PhA showed a negative correlation with serum GDF‐15 concentrations in total subjects. PhA showed a positive correlation with serum albumin (Alb) levels, hemoglobin (Hb) levels, estimated glomerular filtration rate (eGFR), and grip strength, and serum GDF‐15 concentrations showed a negative correlation with serum Alb levels, Hb levels, eGFR, and grip strength. In multivariate regression analysis, after adjusting for age, PhA reflected the association with grip strength. For the presence or absence of muscle weakness measured by handgrip strength, in men, PhA had a cutoff value of 4.35 and an area under the curve (AUC) of 0.857, while GDF‐15 had a cutoff value of 2012 pg/mL and an AUC of 0.773. In women, PhA had a cutoff value of 4.25 and an AUC of 0.804, while GDF‐15 had a cutoff value of 1109 pg/mL and an AUC of 0.764.

**Conclusion:**

PhA showed a negative correlation with serum GDF‐15 concentrations in hospitalized patients with cardiovascular disease. Both PhA and serum GDF‐15 concentrations might be considered as a biomarker of sarcopenia or cachexia.

## 1. Introduction

Malnutrition is well known to be associated with prolonged hospitalization, increased readmission rates, and higher mortality in patients with cardiovascular disease (CVD) [1–5]. Sarcopenia, a progressive loss of skeletal muscle mass and strength commonly seen in older individuals, is also frequently observed in patients with CVD [6–9] and has been shown to increase the risk of mortality, particularly in those with cancer [10].

Phase angle (PhA), a measure derived from bioelectrical impedance analysis, reflects cellular health, cell membrane integrity, and the body’s nutritional and inflammatory status [11–13]. PhA has emerged as a reliable prognostic marker in several diseases, including chronic kidney disease [14], CVD [13], and cancer [15]. Our previous research has shown that PhA is associated significantly with muscle strength in hospitalized patients with CVD and may serve as a useful marker for identifying sarcopenia and malnutrition [16, 17]. Growth differentiation factor (GDF)–15 is a stress‐responsive cytokine that belongs to the transforming growth factor–β superfamily [18]. Elevated GDF‐15 levels have been reported in various pathological conditions, including CVD [19], inflammation [20], cancer [21], and chronic kidney disease [22]. Serum GDF‐15 levels also increase with age [18] and have been found to rise in response to various clinical stressors, such as chronic obstructive pulmonary disease [23], cardiac surgery [19], and critical illness [24]. GDF‐15 expression is closely linked to mitochondrial stress, reflecting its role in diverse physiological and pathological processes [25]. Our previous studies demonstrated a significant association between GDF‐15 concentrations and skeletal muscle mass in older adults with CVD, suggesting the potential utility of GDF‐15 as a biomarker for sarcopenia [26, 27].

GDF‐15 is a multifunctional biomarker associated with disease severity in patients with CVD [19, 26, 27]. Similarly, PhA serves as an indicator of nutritional status and sarcopenia in patients with CVD [13, 16, 17]. As mitochondrial function declines due to aging or disease, cells release GDF‐15 as a stress response [28, 29]. Furthermore, when energy deficiency persists, cells can no longer maintain their membranes, leading to a decrease in PhA [30, 31]. However, no studies to date have evaluated the relationship between PhA and GDF‐15 or explored their combined significance. The aim of this study was to investigate the association between PhA and GDF‐15, along with their regulatory factors, in patients with CVD, including heart failure.

## 2. Methods

### 2.1. Participants

The study included patients with heart failure (*n* = 91), patients undergoing aortic valve replacement (AVR) or transcatheter aortic valve implantation (TAVI) (*n* = 48), and healthy older individuals (*n* = 73) (Table 1). It was approved by the Regional Ethics Committee of Dokkyo Medical University Hospital (approval number: 27077).

**TABLE 1:** Patient characteristics.

	Heart failure (*n* = 91)	TAVI/AVR (*n* = 48)	Healthy (*n* = 73)	Total (*N* = 212)
Age, years	75.0 ± 10.7	80.1 ± 6.3	76.3 ± 6.4	76.6 ± 8.7
Sex (male/female)	57 (63)/34 (37)	12 (25)/36 (75)	6 (8)/67 (92)	75 (35)/137 (65)
BMI, kg/m^2^	21.8 ± 3.7	23.0 ± 3.3	22.7 ± 2.8	22.4 ± 3.3
Alb, g/dL	3.5 ± 0.4	3.8 ± 0.6	4.3 ± 0.2	3.9 ± 0.6
eGFR, mL/min/1.73 m^2^	46.9 ± 17.7	59.9 ± 26.1	67.3 ± 13.1	56.7 ± 20.1
hsCRP, mg/L	0.82 ± 2.02	0.38 ± 0.77	0.14 ± 0.28	0.48 ± 1.42
Hb, g/dL	12.3 ± 2.0	11.6 ± 1.6	13.1 ± 1.2	12.5 ± 1.7
GDF‐15, pg/mL	2795 ± 1647	1632 ± 1135	1041 ± 492	1872 ± 1434
BNP, pg/mL	799 ± 922	333 ± 407	—	—
LVEF, %	38.6 ± 14.2	61.9 ± 11.7	—	—
SMI, kg/m^2^	5.95 ± 1.31	5.55 ± 0.86	6.09 ± 0.64	5.93 ± 1.01
ECW/TBW	0.402 ± 0.015	0.403 ± 0.011	0.397 ± 0.006	0.400 ± 0.012
PhA, °	4.17 ± 0.99	4.16 ± 0.83	4.71 ± 0.48	4.41 ± 0.82
Walking speed, m/s	0.85 ± 0.27	0.86 ± 0.36	1.40 ± 0.31	1.11 ± 0.41
Grip strength, kgf	22.9 ± 9.0	18.2 ± 6.0	23.6 ± 4.9	22.3 ± 7.3

*Note:* Alb, albumin; Hb, hemoglobin; ECW/TBW, extracellular water: total body water ratio; PhA, phase angle. Data are shown as mean ± SD, or number (%).

Abbreviations: AVR = aortic valve replacement; BMI = body mass index; BNP = brain natriuretic peptide; eGFR = estimated glomerular filtration rate; GDF = growth differentiation factor; hsCRP = high‐sensitive C‐reactive protein; LVEF = left ventricular ejection fraction; SD = standard deviation; SMI = skeletal muscle mass index; TAVI = transcatheter aortic valve implantation.

### 2.2. Data Collection

Blood samples were collected after a 12 h fast, and hemoglobin (Hb), serum albumin (Alb), creatinine, and plasma brain natriuretic peptide (BNP) levels were measured; the high‐sensitive C‐reactive protein (hsCRP) level was measured via an immunonephelometric assay. Standard echocardiographic imaging was performed for the evaluation of left ventricular ejection fraction (LVEF) in patients with heart failure and those undergoing AVR or TAVI. Estimated glomerular filtration rate (eGFR) was evaluated using the following equations:

(1) eGFR mL/min/1.73m2=1941.0940.287×serum creatinine−×age− men,

(2) eGFR mL/min/1.73m2=1940.739×serum creatinine−1.094×age−0.287× women.

### 2.3. Enzyme‐Linked Immunosorbent Assay (ELISA)

Serum GDF‐15 levels were measured with the Human GDF‐15 Quantikine ELISA Kit (DGD150 for GDF‐15; R&D Systems, Minneapolis, MN, USA) as previously reported [32]. The mean intraassay coefficient of variation (CV) was 2.3%, and the interassay CV was 5.4%. The detection threshold of GDF‐15 was 2.0 pg/mL.

### 2.4. Bioelectrical Impedance Analyzer (BIA) Measurements

A multifrequency BIA, the In‐Body S10 Biospace device (Biospace Co., Ltd., Seoul, Korea/Model JMW140), was used according to the manufacturer’s guidelines, as described in detail previously [16, 17]. The measurements were carried out while the subjects rested quietly in the supine position, with their elbows extended and relaxed along their trunk. BIA‐derived body components, such as skeletal muscle mass index (SMI), extracellular water: total body water ratio (ECW/TBW), and PhA values, were recorded. Grip strength was measured twice with the right hand, and the higher value was adopted. Walking speed was measured by having patients with CVD walk 4 m and healthy individuals walk 10 m. Sarcopenia was evaluated and defined according to the Asian Working Group for Sarcopenia criteria (men: grip strength < 26 kg or walking speed ≤ 0.8 m/s and SMI < 7.0 kg/m^2^; women: grip strength < 18 kg or walking speed ≤ 0.8 m/s and SMI < 5.7 kg/m^2^) [7]. Muscle weakness was determined as grip strength < 26 kg in men and < 18 kg in women.

### 2.5. Statistical Analysis

Comparisons among the three groups were performed by one‐way analysis of variance if normality was confirmed in all groups or the Kruskal–Wallis test if normality was not confirmed in any group. The Kolmogorov–Smirnov test was used to assess normality. Correlation analysis was performed using the Pearson method if both variables were normally distributed or the Spearman method if either variable was not normally distributed. For multivariate linear regression analysis, variables were log‐transformed if their residuals were not normally distributed, and the number of cases/the number of independent and adjustment variables ≥ 10 was confirmed. A variance inflation factor < 5 was also confirmed to evaluate multicollinearity. Receiver operating characteristic (ROC) curve analysis was performed separately for men and women to test PhA and GDF‐15 for the presence or absence of sarcopenia and muscle weakness. Statistical analysis was performed using SPSS Version 28 for Windows (IBM Corp., New York, NY, USA). A *p* value of < 0.05 was regarded as significant.

## 3. Results

PhA showed a negative correlation with serum GDF‐15 concentrations (*r* = −0.505, *p* < 0.001) in total subjects. PhA showed a positive correlation with serum Alb levels (*r* = 0.504, *p* < 0.001), Hb levels (*r* = 0.441, *p* < 0.001), eGFR (*r* = 0.305, *p* < 0.001), and grip strength (*r* = 0.559, *p* < 0.001). Serum GDF‐15 concentrations showed a negative correlation with serum Alb levels (*r* = −0.607, *p* < 0.001), Hb levels (*r* = −0.381, *p* < 0.001), eGFR (*r* = −0.670, *p* < 0.001), and grip strength (*r* = −0.158, *p* = 0.037).

PhA correlated positively with serum Alb levels, Hb levels, SMI, walking speed, and grip strength in both men and women (Table 2a). PhA correlated negatively with serum GDF‐15 concentrations and ECW/TBW. In women, PhA correlated positively with body mass index (BMI) and eGFR and negatively with age. In both men and women, serum GDF‐15 concentrations showed a positive correlation with serum hsCRP levels and ECW/TBW and a negative correlation with serum Alb levels, eGFR, Hb levels, PhA, walking speed, and grip strength (Table 2b). In women, serum GDF‐15 concentrations showed a positive correlation with age and a negative correlation with BMI and SMI.

**TABLE 2:** Relationship between PhA and serum GDF‐15 concentrations and clinical data.

a: Relationship between PhA and clinical data			
	Men (*n* = 75)	Women (*n* = 137)	Total (*n* = 212)
Age	−0.111 (0.420)	−0.352 (< 0.001)^∗∗∗^	−0.262 (0.001)^∗∗∗^
BMI	0.144 (0.315)	0.491 (< 0.001)^∗∗∗^	0.350 (< 0.001)^∗∗∗^
Alb	0.499 (< 0.001)^∗∗∗^	0.582 (< 0.001)^∗∗∗^	0.504 (< 0.001)^∗∗∗^
eGFR	0.218 (0.137)	0.377 (< 0.001)^∗∗∗^	0.305 (< 0.001)^∗∗∗^
hsCRP	−0.205 (0.150)	−0.123 (0.216)	−0.148 (0.067)
Hb	0.321 (0.020)^∗^	0.511 (< 0.001)^∗∗∗^	0.441 (< 0.001)^∗∗∗^
GDF‐15	−0.617 (< 0.001)^∗∗∗^	−0.579 (< 0.001)^∗∗∗^	−0.505 (< 0.001)^∗∗∗^
SMI	0.281 (0.042)^∗^	0.554 (< 0.001)^∗∗∗^	0.418 (< 0.001)^∗∗∗^
ECW/TBW	−0.881 (< 0.001)^∗∗∗^	−0.885 (< 0.001)^∗∗∗^	−0.808 (< 0.001)^∗∗∗^
Walking speed	0.520 (0.001)^∗∗∗^	0.620 (< 0.001)^∗∗∗^	0.543 (< 0.001)^∗∗∗^
Grip strength	0.723 (< 0.001)^∗∗∗^	0.551 (< 0.001)^∗∗∗^	1.559 (< 0.001)^∗∗∗^

**b: Relationship between serum GDF-15 concentrations and clinical data**			
	**Men (*n* = 75)**	**Women (*n* = 137)**	**Total (*n* = 212)**

Age	−0.039 (0.751)	0.292 (0.001)^∗∗∗^	0.193 (0.006)^∗∗^
BMI	−0.210 (0.123)	−0.196 (0.030)^∗^	−0.174 (0.021)^∗^
Alb	−0.422 (0.001)^∗∗∗^	−0.498 (< 0.001)^∗∗∗^	−0.607 (< 0.001)^∗∗∗^
eGFR	−0.558 (< 0.001)^∗∗∗^	−0.656 (< 0.001)^∗∗∗^	−0.670 (< 0.001)^∗∗∗^
hsCRP	0.309 (0.016)^∗^	0.195 (0.029)^∗^	0.280 (< 0.001)^∗∗∗^
Hb	−0.473 (< 0.001)^∗∗∗^	−0.448 (< 0.001)^∗∗∗^	−0.381 (< 0.001)^∗∗∗^
SMI	−0.072 (0.616)	−0.453 (< 0.001)^∗∗∗^	−0.101 (0.193)
ECW/TBW	0.607 (< 0.001)^∗∗∗^	0.499 (< 0.001)^∗∗∗^	0.480 (< 0.001)^∗∗∗^
PhA	−0.617 (< 0.001)^∗∗∗^	−0.579 (< 0.001)^∗∗∗^	−0.505 (< 0.001)^∗∗∗^
Walking speed	−0.383 (0.016)^∗^	−0.490 (< 0.001)^∗∗∗^	−0.559 (< 0.001)^∗∗∗^
Grip strength	−0.408 (0.002)^∗∗^	−0.434 (< 0.001)^∗∗∗^	−0.158 (0.037)^∗^

*Note:* Alb, albumin; Hb, hemoglobin; ECW/TBW, extracellular water: total body water ratio; PhA, phase angle. Data are shown as *r*‐value (*p* value).

Abbreviations: BMI = body mass index; eGFR = estimated glomerular filtration rate; GDF = growth differentiation factor; hsCRP = high‐sensitive C‐reactive protein; SMI = skeletal muscle mass index.

^∗^ *p* < 0.05.

^∗∗^ *p* < 0.01.

^∗∗∗^ *p* < 0.001.

**FIGURE 1:**
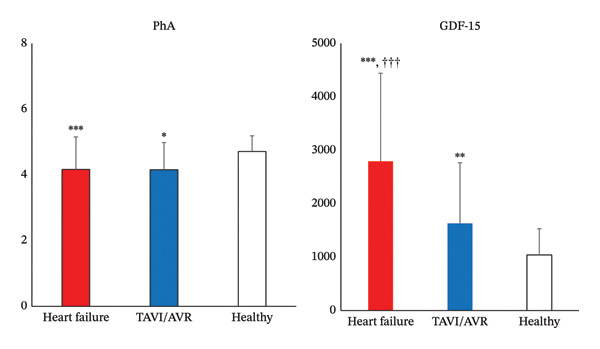
Comparison of phase angle and serum GDF‐15 concentrations among patients with heart failure, TAVI/AVR, and healthy older individuals. TAVI: transcatheter aortic valve implantation; AVR: aortic valve replacement; PhA: phase angle; GDF: growth differentiation factor. ^∗∗^ *p* < 0.01. ^∗∗∗^ *p* < 0.001 vs. healthy subjects. ^†††^ *p* < 0.001 vs. TAVI/AVR.

**FIGURE 2:**
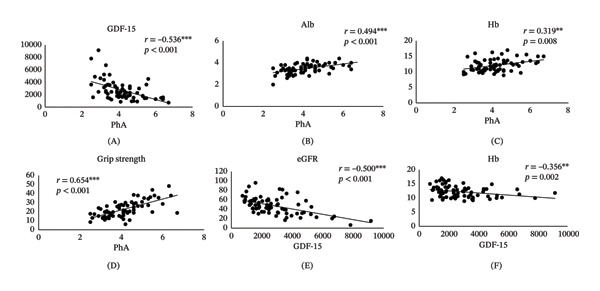
Correlation between clinical data and phase angle and serum GDF‐15 concentrations in patients with heart failure. Correlations between serum GDF‐15 and phase angle (A), serum Alb levels and phase angle (B), Hb levels and phase angle (C), grip strength and phase angle (D), eGFR and serum GDF‐15 concentrations (E), and Hb levels and serum GDF‐15 concentrations (F). Alb: albumin; eGFR: estimated glomerular filtration rate; Hb: hemoglobin; PhA: phase angle; GDF: growth differentiation factor. ^∗∗^ *p* < 0.01. ^∗∗∗^ *p* < 0.001.

**FIGURE 3:**
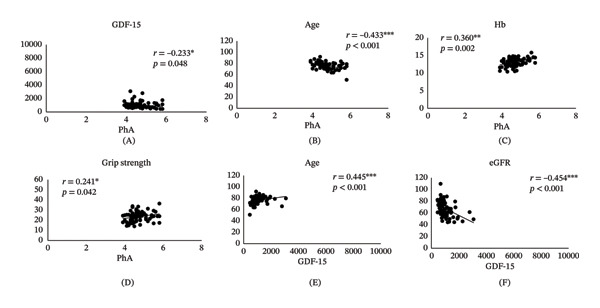
Correlation between clinical data and phase angle and serum GDF‐15 concentrations in healthy older individuals. Correlations between serum GDF‐15 and phase angle (A), age and phase angle (B), Hb levels and phase angle (C), grip strength and phase angle (D), age and serum GDF‐15 concentrations (E), and eGFR and serum GDF‐15 concentrations (F). PhA: phase angle; GDF: growth differentiation factor; Hb: hemoglobin; eGFR: estimated glomerular filtration rate. ^∗^ *p* < 0.05. ^∗∗^ *p* < 0.01. ^∗∗∗^ *p* < 0.001.

In patients with heart failure and patients with AVR or TAVI, PhA was lower, and serum GDF‐15 concentrations were higher compared to healthy older individuals (Figure 1; Table 3). In patients with heart failure, PhA correlated positively with serum Alb levels, Hb levels, and grip strength, and negatively with serum GDF‐15 concentrations. Serum GDF‐15 concentrations correlated negatively with eGFR and Hb levels (Figure 2). In healthy older individuals, PhA correlated positively with Hb levels and grip strength and negatively with serum GDF‐15 concentrations and age. Serum GDF‐15 concentrations correlated positively with age and negatively with eGFR (Figure 3).

**TABLE 3:** Comparison of PhA, serum GDF‐15 concentrations, and clinical data between patients with heart failure and TAVI/AVR and healthy older subjects.

	Heart failure (*n* = 91)	TAVI/AVR (*n* = 48)	Healthy subjects (*n* = 73)	*p* value
PhA,°	4.17 ± 0.99^∗∗∗^	4.16 ± 0.83^∗^	4.71 ± 0.48	< 0.001
GDF‐15, pg/mL	2795 ± 1647^∗∗∗^ ^,^ ^2^	1632 ± 1135^∗∗^	1041 ± 492	< 0.001
Alb, g/dL	3.5 ± 0.4^∗∗∗^ ^,^ ^1^	3.8 ± 0.6^∗∗∗^	4.3 ± 0.2	< 0.001
Grip strength, kgf	22.9 ± 9.0^†^	18.2 ± 6.0^∗∗∗^	23.6 ± 4.9	0.001
Walking speed, m/s	0.85 ± 0.27^∗∗∗^	0.86 ± 0.36^∗∗∗^	1.40 ± 0.31	< 0.001
eGFR, mL/min/1.73 m^2^	46.9 ± 17.7^∗∗∗^ ^,^ ^†^	59.9 ± 26.1	67.3 ± 13.1	< 0.001
Age, years	75.0 ± 10.7^1^	80.1 ± 6.3^∗^	76.3 ± 6.4	0.006

*Note:* Alb, albumin; PhA = phase angle.

Abbreviations: AVR = aortic valve replacement; eGFR = estimated glomerular filtration rate; GDF = growth differentiation factor; TAVI = transcatheter aortic valve implantation.

^∗^ *p* < 0.05.

^∗∗^ *p* < 0.01.

^∗∗∗^ *p* < 0.001 vs. healthy subjects.

^†^ *p* < 0.05.

^1^ *p* < 0.01.

^2^ *p* < 0.001 vs. TAVI/AVR.

In multivariate regression analysis, after adjusting for age, log (GDF‐15) reflected the association with PhA (men: *β* = −0.425, *p* < 0.001; women: *β* = −0.285, *p* = 0.004) and eGFR (men: *β* = −0.460, *p* < 0.001; women: *β* = −0.476, *p* < 0.001), and in men, it also reflected the association with Hb levels (*β* = −0.216, *p* = 0.038) (Table 4a). PhA reflected the association with grip strength (men: *β* = 0.753, *p* < 0.001; women: *β* = 0.272, *p* = 0.003), and in women, it also reflected the association with serum Alb levels (*β* = 0.377, *p* < 0.001) and Hb levels (*β* = 0.202, *p* = 0.018) (Table 4b).

**TABLE 4:** Multivariate linear regression analysis of relationship between serum GDF‐15 level and PhA and clinical data.

a. Relationship between serum GDF‐15 level and PhA and clinical data.		
Dependent variable: log GDF‐15	Model 1men/women	Model 2 men/women
Adjusted *R* ^2^	0.633/0.598	0.644/0.596
Independent variable	β‐value (*p*‐value)	β‐value (*p*‐value)
PhA	−0.404 (< 0.001)^∗∗∗^/−0.263 (0.005)^∗∗^	−0.425 (< 0.001)^∗∗∗^/−0.285 (0.004)^∗∗^
eGFR	−0.502 (< 0.001)^∗∗∗^/−0.474 (< 0.001)^∗∗∗^	−0.460 (< 0.001)^∗∗∗^/−0.476 (< 0.001)^∗∗∗^
Hb	−0.183 (0.074)/−0.182 (0.042)^∗^	−0.216 (0.038)^∗^/−0.176 (0.050)
Alb	‐/−0.106 (0.252)	‐/−0.105 (0.259)

**b. Relationship between PhA and clinical data**		
**Dependent variable: PhA**	**Model 1 men/women**	**Model 2 men/women**

Adjusted *R* ^2^	0.569/0.513	0.571/0.518
Independent variable	β‐value (*p*‐value)	β‐value (*p*‐value)
Grip strength	0.700 (< 0.001)^∗∗∗^/0.330 (< 0.001)^∗∗∗^	0.753 (< 0.001)^∗∗∗^/0.272 (0.003)^∗∗^
Hb	0.079 (0.445)/0.207 (0.016)^∗^	0.111 (0.305)/0.202 (0.018)^∗^
Alb	0.080 (0.496)/0.362 (< 0.001)^∗∗∗^	0.051 (0.671)/0.377 (< 0.001)^∗∗∗^

*Note:* Model 1, multivariate analysis, unadjusted; Model 2, multivariate analysis adjusted by age. Data are shown as β‐value (*p*‐value). Hb, hemoglobin; Alb, albumin; PhA = phase angle.

Abbreviations: eGFR = estimated glomerular filtration rate; GDF = growth differentiation factor.

^∗^ *p* < 0.05.

^∗∗^ *p* < 0.01.

^∗∗∗^ *p* < 0.001.

**FIGURE 4:**
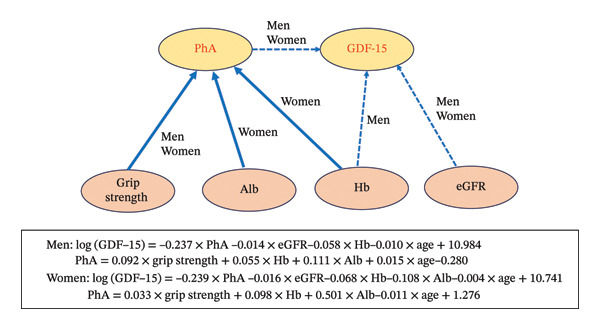
Determinants and estimation formulas for PhA and serum GDF‐15 concentrations in men and women. Solid lines indicate positive correlations, and dashed lines indicate negative correlations. PhA: phase angle; GDF: growth differentiation factor; Alb: albumin; Hb: hemoglobin; eGFR: estimated glomerular filtration rate.

In men, the regression equation for GDF‐15 was log (GDF‐15) = −0.237 × PhA‐0.014 × eGFR‐0.058 × Hb‐0.010 × age + 10.984 (Figure 4). The regression equation for PhA was obtained as PhA = 0.092 × grip strength + 0.055 × Hb + 0.111 × Alb + 0.015 × age‐0.280. For women, the regression equation for GDF‐15 was log (GDF‐15) = −0.239 × PhA‐0.016 × eGFR‐0.068 × Hb‐0.108 × Alb‐0.004 × age + 10.741. The regression equation for PhA was PhA = 0.033 × grip strength + 0.098 × Hb + 0.501 × Alb‐0.011 × age + 1.276.

**FIGURE 5:**
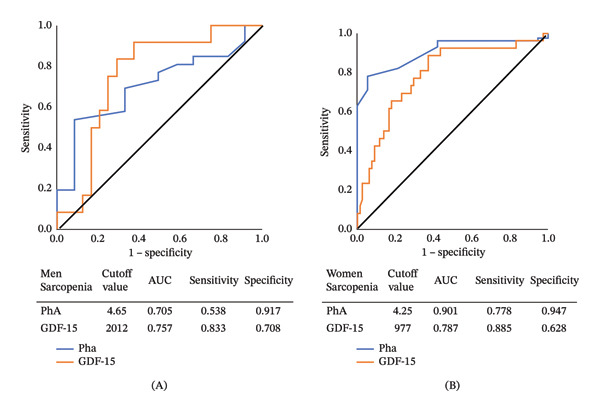
ROC curve analysis of PhA and GDF‐15 for sarcopenia in men (A) and women (B). ROC: receiver operating characteristic; PhA: phase angle; AUC: area under the curve.

In ROC curve analysis for the presence or absence of sarcopenia, for men, PhA had a cutoff value of 4.65, an area under the curve (AUC) of 0.705, a sensitivity of 53.8%, and a specificity of 91.7%, while serum GDF‐15 concentrations had a cutoff value of 2012 pg/mL, an AUC of 0.757, a sensitivity of 83.3%, and a specificity of 70.8% (Figure 5). In women, PhA had a cutoff value of 4.25, an AUC of 0.901, a sensitivity of 77.8%, and a specificity of 94.7%, while serum GDF‐15 concentrations had a cutoff value of 977 pg/mL, an AUC of 0.787, a sensitivity of 88.5%, and a specificity of 62.8%.

**FIGURE 6:**
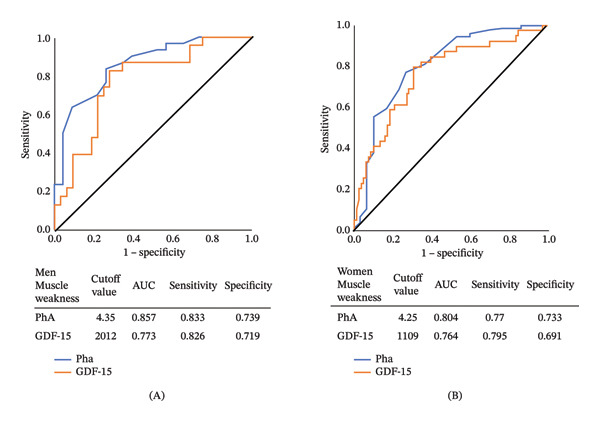
ROC curve analysis of PhA and GDF‐15 for muscle weakness in men (A) and women (B). ROC: receiver operating characteristic; PhA: phase angle; AUC: area under the curve.

For the presence or absence of muscle weakness, in men, PhA had a cutoff value of 4.35, an AUC of 0.857, a sensitivity of 83.3%, and a specificity of 73.9%, while serum GDF‐15 concentrations had a cutoff value of 2012 pg/mL, an AUC of 0.773, a sensitivity of 82.6%, and a specificity of 71.9% (Figure 6). In women, PhA had a cutoff value of 4.25, an AUC of 0.804, a sensitivity of 77.0%, and a specificity of 73.3%, while serum GDF‐15 concentrations had a cutoff value of 1109 pg/mL, an AUC of 0.764, a sensitivity of 79.5%, and a specificity of 69.1%.

## 4. Discussion

In this study, PhA showed a negative correlation with serum GDF‐15 concentrations. PhA showed a positive correlation with serum Alb levels, Hb levels, eGFR, and grip strength, and serum GDF‐15 concentrations showed a negative correlation with serum Alb levels, Hb levels, eGFR, and grip strength. In addition, in gender‐stratified multivariate regression analysis, after adjusting for age, PhA reflected the association with grip strength in both men and women, and in women, serum Alb levels and Hb levels were also identified as associated factors. ROC curve analysis showed that both PhA and serum GDF‐15 concentrations might be considered as a biomarker of sarcopenia or cachexia.

PhA is derived from the relationship between resistance and reactance [33, 34]. Physiologically, PhA depends on cell membrane integrity and water distribution between intracellular and extracellular spaces [11], and low values may reflect impaired cellular integrity or malnutrition [35]. Also, PhA is associated with poor prognosis in many disease groups, including patients with heart failure [36] and patients receiving dialysis [37]. Colín‐Ramírez and colleagues [36] showed that in patients with heart failure, those with PhA below the lowest quartile range (< 4.2) had lower mean BMI, grip strength, and Hb levels, and the highest proportions of New York Heart Association Class III disease and renal failure. They also showed via Cox regression analysis that PhA < ° 4.2° was an independent prognostic factor for mortality, even after adjusting for age, Hb levels, and diabetes. Another study showed that PhA values correlated positively with serum Alb–related nutritional indicators, prealbumin, fat‐free mass, and mid‐upper arm muscle circumference, and in multivariate logistic regression analysis, PhA was a predictive factor in patients undergoing maintenance dialysis [37].

A recent study demonstrated that PhA correlates with physical function and independently predicts long‐term mortality after cardiovascular surgery [38]. Regarding the association between PhA and physical function, PhA has been shown to be associated with age, sex, BMI, comorbidities, and frailty as measured by the Short Physical Performance Battery and Fried score in patients undergoing cardiac surgery [13]. In our previous studies, we analyzed body composition and nutritional markers using the BIA method in hospitalized patients with CVD [16, 17]. Gender‐stratified multivariate analysis demonstrated that grip strength was an independent predictor of PhA after adjusting for BMI and age [16]. Also, gender‐stratified multivariate regression analysis showed that when ECW/TBW was 0.4 or higher, PhA was a stronger independent determinant of grip strength and logarithmic thigh muscle thickness than SMI. Thus, PhA was suggested to be a good marker of age‐related sarcopenia or disease‐related cachexia in patients with CVD, including chronic heart failure [17]. Furthermore, the cutoff values for sarcopenia and cachexia reported in previous studies (4.2°–4.6°) [16, 37, 38] were generally consistent with the values identified in the present study, reaffirming the clinical utility of PhA.

GDF‐15 is known to be produced under various stressful conditions, such as inflammation [20] and oxidative stress [39], and is an independent predictor of mortality in individuals with heart failure [18] and older individuals [40]. The present study showed that PhA showed a negative correlation with serum GDF‐15 concentrations in hospitalized patients with CVD. Results from studies in both humans and animal models have found that the systemic inflammatory response is involved in the development of critical illness–associated weakness [41]. Tumor necrosis factor (TNF)–α promoted skeletal muscle atrophy with reduced protein content and reduced myotube diameter via nuclear factor‐κB [42]. In addition, TNF‐α impaired muscle function (i.e., force per unit of muscle) through oxidative stress [43], and interleukin (IL)‐1 impaired it through reduction in sarcoplasmic reticulum calcium release [44]. IL‐6 may also lead to loss of muscle mass via an indirect pathway by interfering with the signaling of insulin‐like growth factor (IGF)‐1, an important anabolic promoter [45]. GDF‐15 has also been identified as a mediator of critical illness–related muscle atrophy [24]. Therefore, it was suggested that inflammation mediated by cytokines such as TNF‐α and GDF‐15 is associated with the relationship between PhA and physical function in hospitalized patients with CVD.

In our study, multivariate regression analysis showed that logarithmic GDF‐15 reflected the association with PhA, and PhA reflected the association with grip strength after adjusting for age. Therefore, factors other than age were thought to be determinants of serum GDF‐15 concentrations, so it was probably cachexic in our cardiovascular patients. Our previous study showed that serum GDF‐15 level was positively correlated with serum TNF‐α level, and a correlation was observed between TNF‐α and muscle mass and grip strength in men in preoperative cardiovascular surgery patients [32]. Moreover, IGF‐1 decreased with age and was regarded as a potential mediator of sarcopenia or frailty [46]. That study also showed a negative correlation between GDF‐15 and IGF‐1 concentration, which was observed only in women, but not in men [32]. Also, cardiac cachexia and sarcopenia have been reported to lead to increased levels of adiponectin in heart failure [47–49]. Another one of our previous study showed that patients with a high serum adiponectin level (> 6.2 μg/mL, the cutoff value of the ROC curve) had lower Hb, LVEF, grip strength, knee extension strength, anterior mid‐thigh muscle thickness, and SMI, compared to those with a low serum adiponectin level (< 6.2 μg/mL) in preoperative cardiovascular surgery patients [50]. In addition, GDF‐15 has been reported to reflect energy metabolism disorders caused by mitochondrial dysfunction [28]. Kemp et al. [51] showed that preexisting mitochondrial dysfunction and reduced cortisol inactivation contribute to muscle loss following aortic surgery in men, and the data also implicate GDF‐15 and IL‐15 receptor subunit alpha (IL‐15Rα) in mitochondrial dysfunction. Therefore, it is possible that inflammation, adiponectin, and mitochondrial dysfunction are involved in the association between GDF‐15 and cachexia or sarcopenia observed in the present study.

On the other hand, PhA is influenced by the structural integrity of the cell membrane, intracellular water content, and body cell mass. Hirano et al. [30] demonstrated the association between walking speed and PhA and muscle quality in older adults. Besides, individuals with healthy cell membranes have higher PhA, while PhA decreases when energy deficiency persists, and cell membranes are damaged due to aging, disease, or malnutrition [30, 31]. In the present study, we showed that both PhA and serum GDF‐15 concentrations might be considered as a biomarker of sarcopenia or cachexia.

This study has several limitations. First, there was a statistically significant age difference among the three groups (heart failure, TAVI/AVR, and healthy; *p* = 0.006). Given the significant impact of serum GDF‐15 levels and PhA on age, the presence of this group difference in baseline demographics might introduce a confounding variable. Further expansion of the sample size is needed to allow for analysis across the three groups (heart failure, TAVI/AVR, and healthy). Second, upon examination of the ROC curve analysis, it is found that for the prediction of sarcopenia in women, the specificity of GDF‐15 was only 62.8%. A specificity of 62.8% is not particularly useful as a standalone diagnostic test because of the high rate of false positives.

## 5. Conclusions

The present study demonstrated that PhA showed a negative correlation with serum GDF‐15 concentrations in hospitalized patients with CVD. It also showed that both PhA and serum GDF‐15 concentrations might be considered as a biomarker of sarcopenia or cachexia.

## Funding

JSPS KAKENHI, 19H03981, 20K11259, 22H03457, 24K14354.

## Conflicts of Interest

The authors declare no conflicts of interest.

## Data Availability

The data that support the findings of this study are available from the corresponding author upon reasonable request.
